# Relationship between Human Immunodeficiency Virus (HIV) Knowledge, HIV-Related Stigma, and HIV Testing among Young Black Adults in a Southeastern City

**DOI:** 10.3389/fpubh.2017.00047

**Published:** 2017-03-13

**Authors:** Eunice Okumu, David H. Jolly, Le’Marus Alston, Natalie T. Eley, Michelle Laws, Kathleen M. MacQueen

**Affiliations:** ^1^Global Health Research Department, FHI 360, Durham, NC, USA; ^2^Department of Public Health and Education, North Carolina Central University, Durham, NC, USA; ^3^North Carolina Wesleyan College, Rocky Mount, NC, USA; ^4^Virginia Commonwealth University School of Medicine, Richmond, VA, USA

**Keywords:** HIV knowledge, HIV-related stigma, HIV testing, HIV/AIDS information, young Black adults

## Abstract

The southeast is identified as the epicenter of the nation’s human immunodeficiency virus (HIV) epidemic, accounting for nearly 44% of all persons living with a HIV diagnosis in the United States. HIV stigma and knowledge have been cited as some of the complex factors increasing risk of acquiring HIV within African-American communities. We sought to understand how HIV knowledge and HIV-related stigma impact HIV testing experience among young Black adults who completed a community-based participatory research survey in a Southeastern city. Survey measures were developed with active engagement among the research team and community members, with the goal of balancing community knowledge, interests and concerns with scientific considerations, and the realities of funding and the project timeline. A total of 508 of the 513 audio computer-assisted self-interview questionnaires completed were analyzed. Eighty-one percent of participants had ever tested and had an intention-to-test for HIV in the next 12 months. Overall, analyses revealed low HIV-related stigma and relatively moderate to high HIV knowledge among young Black adults in the Southeastern city. Logistic regression indicated that having ever tested for HIV was positively correlated with HIV knowledge [odds ratio (OR): 1.50; 95% confidence interval (CI): 1.23–1.84, *p* < 0.001], but inversely correlated with low HIV-related stigma (OR: 0.08; 95% CI: 0.01–0.76, *p* < 0.03). However, there were no significant relationships between HIV-related stigma, HIV knowledge, and intention-to test for HIV in the future. These findings suggest that reducing HIV-related stigma and increasing HIV knowledge are not sufficient in promoting HIV testing (i.e., intention-to-test) among young Black adults in this city, unless specific emphasis is placed on addressing internalized HIV-related stigma and misperceptions about HIV prevention and control.

## Introduction

Blacks account for almost half (44% in 2010) of all new human immunodeficiency virus (HIV) infections in the United States as well as more than one-third (40% in 2013) of all people living with HIV (1). The Centers for Disease Control and Prevention (CDC) estimate that approximately 1 in 20 black men will be diagnosed with HIV during his lifetime, as will 1 in 48 black women (1). Nearly, one-third of ongoing transmission in the United States is attributed to persons unaware of their HIV infection (2). Fourteen percent of the estimated 1.2 million persons living with HIV in the United States in 2011 had undiagnosed infections (2). As of December 31, 2014, the 3-year (2012–2014) average rate of diagnosed HIV infection in North Carolina was 13.7 per 100,000 population (3). In 2014, 1,351 (13.4 per 100,000 population) new diagnoses of HIV infection were reported in North Carolina (3). Of the new infections, 1,341 (16.3 per 100,000 population) infections occurred in the adult/adolescent population, with Black/African-Americans representing 64% of all adult/adolescent infections in North Carolina, with a rate of 48.7 per 100,000 adult/adolescent population (3). Durham County (23.5 per 100,000 population) ranked third highest among the 100 North Carolina counties in the 3-year (2012–2014) average rate of newly diagnosed HIV infection (3).

Research findings suggest that greater HIV knowledge is associated with greater likelihood of HIV testing, however, some studies have either failed to replicate this relationship or found that more knowledge is associated with greater risk-taking (4). HIV-related stigma is documented as a barrier to HIV testing uptake in resource limited countries particularly with reduced access to health care services, reducing the effectiveness of HIV-preventative and treatment behavioral interventions (5, 6). The current study utilized cross-sectional data from a community-based participatory research study that was conducted in Durham County, North Carolina (7), to examine whether HIV-related stigma and knowledge impact HIV testing among young Black adults.

## Materials and Methods

### Study Design and Sampling Strategy

This analysis utilized cross-sectional survey data from the LinCS 2 Durham project, a community-based participatory research study (7–9). The survey was completed by self-identifying Black young adults aged 18–30 years, living in Durham County, North Carolina during the previous 6 months and reporting vaginal or anal sex in the past 6 months. The final study sample included 513 Black young adults who were screened, consented, and enrolled into the study from May 11, 2011 to June 9, 2012. Further details on study design and sampling method have been reported elsewhere (7). The survey measures were developed in collaboration with the project’s Collaborative Council (CC) members, which consisted of a cross-representation of partners, including advocates and policy-makers in allied fields, civil society and grassroots community stakeholders, potential research participants, researchers and sponsors, and program managers for HIV and allied service areas. Of note, the CC members rephrased, deleted, and added some HIV-related stigma and HIV knowledge scale items to create more appropriate/acceptable language for our participants. The changes reflect a local emphasis on direct language, summed up in a LinCS 2 Durham tagline “No bull, just real talk.” The reference to “bull” reflects both the slang meaning as well as the bull as a long-standing symbol for the city of Durham. The revision process began with a total of 20 HIV knowledge and 21 HIV-related stigma validated scale items. A final set of 8 HIV knowledge and 13 HIV-related stigma items were adapted for use in this cross-sectional survey as described in the measures section below. The survey was piloted with the CC members, who then referred about a dozen people meeting survey eligibility criteria to participate in further piloting of the survey to assess clarity, length, and appropriateness to the target population. All study-related documents were reviewed and approved by institutional review boards at FHI 360 (Protection of Human Subjects Committee) and North Carolina Central University (NCCU Institutional Review Board). Participants received written informed consent and were enrolled into the study only after signing the informed consent forms.

### Measures

The survey was administered using audio computer-assisted self-interview on tablet computers and required approximately 45 min to complete. The following provides an overview of the measures included in this analysis.

#### Sociodemographic Characteristics

Participants were asked about education level attained, gender identity, employment status, income, housing, and incarceration (7). Age and residence were determined by verification of identification document(s) (i.e., driver’s license and/or school ID) during the eligibility screening process.

#### HIV/AIDS Information

Participants were asked to identify their main source of HIV/AIDS information. They were given a list of options and told to select all that applied to them: media, health professionals, school, friends/family, church, work place, volunteer or fraternal group, and some other source (closed ended response option).

#### HIV Knowledge

An eight item test was used to assess participants’ knowledge and misperceptions about HIV/AIDS. Items were adapted from measures reported by Carey et al., Carey and Schroder, as well as a survey administered by Kaiser Family Foundation (10–12). As noted above, CC members rephrased, deleted, and added some items to create more appropriate/acceptable language for our participants. To evaluate HIV knowledge and misperceptions, response options including “yes,” “no,” and “refuse to answer” were provided for each of the eight items (see Table 1 for knowledge items). A score of 1 was assigned for correct answers and 0 for incorrect answers and refusals. Correct answers were summed to generate an overall score for each participant (13). High scores indicated high HIV knowledge.

**Table 1:** **Derivation of human immunodeficiency virus (HIV) knowledge items with correct responses noted and descriptive statistics**.

Original items	Revised/final survey item	Total (*n* = 508)	% with correct response	Mean (SD) (mean—scale of 0–1)	*p* Value
When you think about HIV/AIDS, which group of people do you think of first as those who are most likely to be infected? And which group of people do you think of next? Gay men/homosexuals/bisexuals; drug users; teenagers/young people/promiscuous people, etc.^10^	Is it true that only homosexuals get HIV? *(No)*	508	99.4	0.99 (0.08)	0.48
Athletes who share needles when using steroids can get HIV from the needles.^8^	Is it true that a person can get HIV by sharing drug needles? *(Yes)*	508	99.3	0.99 (0.10)	0.54
A person can get HIV by sitting in a hot tub or a swimming pool with a person who has HIV;^9^ a person can get HIV from a toilet seat;^8^ a person can get HIV by sharing a glass of water with someone who has HIV.^8^	Can a person get HIV through casual contact with someone who has HIV? For example, by hugging, sharing a drinking glass, touching a toilet seat, or swimming in a pool? *(No)*	508	93.4	0.97 (0.17)	0.77
You can usually tell if someone has HIV by looking at them.^8^	It is true that you can tell if someone has HIV or AIDS just by the way he or she looks? *(No)*	508	96.3	0.94 (0.24)	0.31
There are drugs available which can lengthen the lives of people who have HIV and AIDS.^10^	If a person has HIV, are there drugs that can keep them healthy and alive for a long time? *(Yes)*	508	91.2	0.91 (0.28)	0.91
A pregnant woman who has HIV can take certain drugs to reduce the risk of her baby being born infected.^10^	If a pregnant woman has HIV, are there drugs she can take to protect her baby from being born with HIV? *(Yes)*	508	71.1	0.66 (0.48)	0.01
There is a vaccine that can stop adults from getting HIV.^8,9^	Is there a vaccine available to protect people from getting infected with HIV? *(No)*	508	82.4	0.80 (0.40)	0.20
There are drugs available that can cure HIV and AIDS/there is a cure for AIDS.^8,10^	Are there drugs that can cure HIV and AIDS? *(No)*	508	86.8	0.82 (0.38)	0.003

*The superscript numbers are citations to the original-scale items*.

#### HIV Experience

In order to understand participants’ personal relevance of HIV/AIDS, they were asked a single question on whether they personally knew anyone who currently had AIDS, had died from AIDS, or had tested positive for HIV (14). Response options including “yes,” “no,” and “refuse to answer” were provided for this question. A score of 1 was given for “yes” response and a 0 for “no” and “refuse to answer.”

#### HIV Stigma

Thirteen items used in this survey were adapted from the AIDS-Related Stigma Scales reported by Kalichman et al. (15, 16), Van Rie et al. (17), and the National AIDS Programs core indicators (18). These items included various attitudes toward people living with HIV/AIDS (PLWHA) and internalized HIV-related stigma (see Table 2 for stigma items). The final items reflect the revisions by the CC members to create more appropriate/acceptable language for our participants. Participants’ responses to HIV-related questions were measured on a four-point Likert scale ranging from 1 (strongly agree) to 4 (strongly disagree). Positively worded questions were reverse coded prior to analysis, and then all scores were summed (15) and averaged by the number of items (19) answered (i.e., non-missing items) to generate an overall score for each participant. Higher scores indicated low HIV-related stigma.

**Table 2:** **Derivation of HIV-related stigma items and descriptive statistics**.

Original items	Revised/final survey item	Total (*n* = 508)	% with positive attitude	Mean (SD) [mean (scale of 1–4)]	*p* Value
People who have AIDS are cursed.^12^	I believe that HIV/AIDS is a curse from God *(disagree/strongly disagree)*	503	94.0	3.53 (0.67)	0.02

A person with AIDS must have done something wrong and deserves to be punished.^12^	I think that people who get HIV/AIDS did something to deserve it *(disagree/strongly disagree)*	507	94.7	3.58 (0.64)	0.001
N/A	People with HIV/AIDS should get the same level of medical care as a person who has cancer or another type of chronic disease *(agree/strongly agree)*	507	95.8	3.63 (0.65)	0.20

I hide my HIV status from others.^13^/If a member of your family became infected with HIV, would you want it to remain a secret?^14^	It is better for the community if a person tells others if they have HIV *(agree/strongly agree)*	507	80.9	3.29 (0.90)	0.20

Some people who have AIDS lose friends when they share with them they have AIDS.^15^	If a friend of mine confided that they were HIV positive, I would cut them off. That friendship would be over *(disagree/strongly disagree)*	508	96.4	3.68 (0.60)	0.002

If a person has AIDS, some community members will behave differently toward that person for the rest of his or her life.^15^	If I told my best friend that I was HIV positive, my friend would not treat me any differently *(agree/strongly agree)*	508	72.1	3.02 (0.99)	0.001

Some people who have AIDS worry that others will reveal their secret.^15^	If I found out that I was HIV positive, it would affect my social life, even if I never told anyone *(disagree/strongly disagree)*	507	23.7	1.88 (1.00)	0.48

I sometimes feel worthless because I am HIV positive.^13^	If I tested positive for HIV, I do not think my life would change very much *(agree/strongly agree)*	507	20.1	1.80 (0.94)	0.55

Some people who have AIDS are afraid that other people in the community will talk about them having AIDS.^15^	I am afraid to talk about HIV because I wouldn’t want anybody to think that I have it *(disagree/strongly disagree)*	508	92.0	3.42 (0.73)	0.14

If a member of your family became sick with HIV, would you be willing to care for him or her in your household?^13^	If I told my family that I had HIV, they would embrace me, support me, and be there for me *(agree/strongly agree)*	508	89.6	3.5 (0.76)	0.23

If a member of your family became sick with HIV, would you be willing to care for him or her in your household?^13^	If I told members of my church or religious group that I had HIV, they would support me and be there for me *(agree/strongly agree)*	507	83.1	3.23 (0.86)	0.19

Some people feel uncomfortable being near those with AIDS.^15^	If my brother or sister were HIV positive, I could never feel the same about them or be comfortable around them *(disagree/Strongly disagree)*	508	95.1	3.71 (0.62)	0.42

Some people prefer not to have those with AIDS living in their community.^15^	If any of my neighbors were HIV positive, they should be forced to move somewhere else *(disagree/strongly disagree)*	508	98.6	3.73 (0.51)	0.14

*The superscript numbers are citations to the original-scale items*.

#### HIV Testing Experience

Participants were asked whether they had ever been tested for HIV, and those reporting they had were asked how long it had been since their last HIV test and where they had received their last test (20). All participants were asked whether they expected to have an HIV test in the next 12 months, excluding blood donations (20).

### Statistical Analyses

All analyses examined differences by gender. As a result, four participants with missing or contradictory gender responses, and another participant who identified as transgender were excluded, resulting in responses from 508 participants which were analyzed using SPSS for windows, version 17.0. Descriptive statistics were used to summarize demographic characteristics, HIV testing experience/intention, and sources of HIV/AIDS information. Categorical variables were created using the 8 HIV knowledge and 13 HIV-related stigma items. The scores for HIV knowledge ranged from 3 to 8, and the mean and median were 7.09 (SD = 1.01) and 7, respectively. The scores for HIV-related stigma ranged from 2 to 4; the mean score was 3.23 (SD = 0.32), and the median was 3. The knowledge score was dichotomized based on the median score: those scoring less than the median score (knowledge scores < 7) were categorized into “low” and those scoring equal to or more than the median score (knowledge scores ≥ 7), were categorized into “high” HIV knowledge (21). The knowledge score was dichotomized in this way because there is no standard cutoff for a critical level of knowledge for the HIV knowledge items used in this survey. The stigma score was dichotomized based on the midpoint of the 4-point Likert scale used; therefore, those with a summary score of <2.5 were categorized into high HIV-related stigma and those with a summary score of ≥2.5 were categorized into low HIV-related stigma. We dichotomized stigma this way to divide the scores into categories of overall agreement or disagreement with the questions asked. Bivariate analyses were conducted to describe and test for differences in participant characteristics by the knowledge and stigma score levels, calculating unadjusted odds ratios (ORs) and 95% confidence intervals (CIs). Logistic regression models were used to determine the association between HIV testing experience (response) and HIV knowledge and HIV-related stigma (predictors, both in separate models). These models included sociodemographic variables (gender, age, and level of education) that were considered potential confounders of the relationships of interest.

## Results

Fifty-three percent of participants self-identified as females, almost half (42.9%) had some college education, 80.5% reported ever testing for HIV, and 81.5% reported an intention-to-test for HIV in the next 12 months. Overall, more female than male (86.4 vs. 73.6%) participants reported testing for HIV in the past and knowing someone who has AIDS, has died from AIDS, or has tested positive for HIV (60.1 vs. 30.9%).

Media such as radio, television, newspapers, and internet were the most commonly cited sources of HIV/AIDS information for the majority of participants (87.8%), while church and other religious institutions were the least frequently cited common sources of information (25.8%). Overall, 77.2% of participants had high HIV knowledge scores and 99.2% participants had low HIV-related stigma. The odds of ever testing for HIV among participants who cited health professionals (OR: 0.54; 95% CI: 0.32–0.93, *p* < 0.03), and church (OR: 0.45; 95% CI: 0.24–0.86, *p* < 0.02) was lower than those who cited school (OR: 2.32; 95% CI: 1.28–4.21, *p* < 0.01) as a source of HIV/AIDS information. The odds ratio of intention-to-test for HIV in the future was higher among participants who cited media (OR: 3.83; 95% CI: 1.40–10.45, *p* < 0.01) as a source of HIV/AIDS information; no other sources were significantly correlated with intention-to-test.

The full models in Tables 3 and 4 show that the odds of ever testing for HIV was higher among participants with high HIV knowledge, but lower among participants with low HIV-related stigma. However, there were no significant relationships between HIV-related stigma, HIV knowledge, and intention-to-test for HIV in the future.

**Table 3:** **Correlations between HIV-related stigma, HIV testing experience, and intention-to-test for HIV**.

	All participants (*n* = 508)	Odds ratios (ORs)	Confidence intervals (95% CIs)	*p* Value
Ever tested for HIV	508	0.08	0.01–0.76	0.03
Intention-to-test for HIV	504	0.22	0.03–1.06	0.14

**Table 4:** **Correlations between HIV knowledge, HIV testing experience, and intention-to-test for HIV**.

	All participants (*n* = 508)	Odds ratios (ORs)	Confidence intervals (95% CIs)	*p* Value
Ever tested for HIV	508	1.50	1.23–1.84	0.001
Intention-to-test for HIV	504	1.22	0.987–1.50	0.07

## Discussion

To accelerate progress toward reducing undiagnosed HIV infections, the CDC and its partners have pursued an approach of expanded HIV testing in communities with high HIV infection rates, recommending that adolescents and adults be tested for HIV infection at least once and that persons at increased risk for HIV infection be tested at least annually (2). As previously noted, Durham County ranked third highest among the 100 North Carolina Counties in the 3-year (2012–2014) average rate of newly diagnosed HIV infection (3). In Durham as elsewhere in the southern United States, young Black adults are among those at highest risk for HIV (3). In this analysis, we explored the relationship between HIV testing, HIV-related stigma, and HIV knowledge among Black young adults in Durham.

Inadequate health literacy, including low HIV knowledge, decreases the likelihood of engaging in preventative health behaviors (4). In this study, HIV knowledge for all participants was high in some areas (especially with regard to HIV transmission) and somewhat lower in others (mostly in prevention and control) (Table 1). Participants with high HIV knowledge scores were more likely to be those who had Bachelor’s education and higher (84.4%), females (81.3%), 26- to 30-year olds (77.6%), and those who selected media (68.0%) as their source of HIV/AIDS information (table not shown). Of note, our findings point to church and other religious institutions as one of the least accurate sources of HIV/AIDS information for Black young adults in Durham. Other studies have found that Black community members often view church leaders as reliable sources of information and support (22). As a result, churches have the potential to play a much larger role in health promotion and disease prevention, particularly HIV/AIDS prevention programs. Since high HIV knowledge was positively associated with HIV testing, it is reasonable to assume that participants who possess more accurate knowledge about HIV transmission, prevention, and control, feel more capable of reducing their risk for contracting HIV by seeking opportunities to know their status. People who are aware of their infection can also make behavioral changes and seek effective treatment to reduce transmission (2).

Overall, our participants had a positive attitude toward PLWHA. In other studies, negative attitude toward PLWHA was associated with a decreased willingness to seek out and attend to information about HIV prevention, diagnosis, and treatment (4). Our participants’ attitude toward PLWHA may help explain why they seek out HIV/AIDS information from the media. However, it is also important to note that a majority of participants (approximately 80%) indicated internalized HIV-related stigma in responses to questions such as “*if I tested positive for HIV, I do not think my life would change very much*” and “*if I found out that I was HIV positive, it would affect my social life, even if I never told anyone*” (Table 2). About half (51.2%) of our participants reported knowing someone who has AIDS, has died from AIDS, or has tested positive for HIV. The positive attitude toward PLWHA together with internalized stigma could be an indication that young Black adults in this community reject stigma directed toward PLWHA, but are also unsure of being accepted in the community should they test positive. In other studies, keeping one’s diagnosis a secret was found to be likely to restrict access to support in coping with and managing one’s illness (23).

About a fifth of the participants in this study said they had never been tested for HIV and, of these, almost all (97%) reported low HIV-related stigma. The most common reasons for not testing are reported elsewhere (7), and included not believing that they were at risk of getting HIV (49%), no desire to get tested (39%), not knowing how or where to go for the test (27%), lack of time to go for the test (18%), and fear of losing family or friends in case of a positive test result (11%). However, almost half (40%) of those who had internalized HIV-related stigma reported never testing for HIV, and approximately 36% reported not having an intention-to-test for HIV in the future. This could be an indication that participants who reported not having a desire to get tested and those who reported fear of losing their social circle (i.e., family or friends) in case of a positive test result might have been discouraged from testing due to internalized stigma.

The current study used HIV knowledge and HIV-related stigma scale items that were revised and piloted in collaboration with the CC members to assess clarity, length, and appropriateness to the target population within the community. However, factor analysis on the revised scale items in order to determine reliability and internal consistency prior to incorporating them into the survey was not conducted. Additionally, the scale items were revised mostly by both the research team and community members who were older than the target age-group. This reflected time constraints and funding limitations that are common with community-based participatory research. As a result, the revisions might have had an impact on how participants responded to the HIV-related stigma and HIV knowledge items. We recommend validating the scale items prior to incorporating them in subsequent research studies. This could include a first round of un-rotated factor analysis in order to determine the number of factors underlying HIV-related stigma or HIV knowledge, and subsequent rotated rounds to provide the relationship among items for each factor. Overall, participants answered the majority of the questions, resulting in sufficient data to enable us draw correlations between HIV testing experience and both HIV-related stigma and HIV knowledge.

## Conclusion

Human immunodeficiency virus testing is a critical gateway to ending the HIV epidemic in the United States and globally, whether by identifying those infected and connecting them to treatment and care, or by bridging those at risk with effective prevention and resources to reduce their likelihood of future infection. Addressing barriers to testing requires understanding of local community dynamics. Our study among young Black adults in a Southeastern United States city highlights the importance of understanding HIV knowledge, where such knowledge is obtained, and how internalized HIV-related stigma influence HIV testing experience and intentions. The findings from this study can also inform HIV testing and linkage to care interventions of the importance of incorporating age and gender specific educational strategies and developing measures to assess gender-based changes in internalized stigma and HIV knowledge. Collaboration efforts with community stakeholders, particularly Black churches could improve the reach of HIV prevention messages and services. Addressing internalized HIV-related stigma and HIV prevention and control misperceptions could help facilitate increased HIV testing among young Black adults in this city.

## Author Contributions

EO made substantial contribution to onsite data collection and management, analysis and interpretation of data, and writing of the initial draft of the manuscript. DJ substantially contributed to the study conception and design, onsite data collection and management, as well as interpretation of data. LA made substantial contribution to data collection and interpretation. NE and ML made substantial contribution to the study design and interpretation of data. KM made substantial contribution to the study conception and design, data collection and management, and data interpretation. All the authors critically reviewed the manuscript for important intellectual content, approved the final version to be published, and agreed to be accountable for all aspects of the work.

## Conflict of Interest Statement

The authors declare that the research was conducted in the absence of any commercial or financial relationships that could be construed as a potential conflict of interest. The reviewer JH and handling editor declared a current collaboration and the handling editor states that the process nevertheless met the standards of a fair and objective review.
