# Corrigendum: Relationship between Human Immunodeficiency Virus (HIV) Knowledge, HIV-Related Stigma, and HIV Testing among Young Black Adults in a Southeastern City

**DOI:** 10.3389/fpubh.2017.00166

**Published:** 2017-07-11

**Authors:** Eunice Okumu, David H. Jolly, Le’Marus Alston, Natalie T. Eley, Michelle Laws, Kathleen M. MacQueen

**Affiliations:** ^1^Global Health Research Department, FHI 360, Durham, NC, United States; ^2^Department of Public Health and Education, North Carolina Central University, Durham, NC, United States; ^3^North Carolina Wesleyan College, Rocky Mount, NC, United States; ^4^Virginia Commonwealth University School of Medicine, Richmond, VA, United States

**Keywords:** human immunodeficiency virus knowledge, human immunodeficiency virus-related stigma, human immunodeficiency virus testing, human immunodeficiency virus/AIDS information, young Black adults

Error in Table

In the original article, there was a mistake in **TABLE 1 | Derivation of human immunodeficiency virus (HIV) knowledge items with correct responses noted and descriptive statistics and TABLE 2 | Derivation of HIV-related stigma items and descriptive statistics** as published. The citations of scale items were misplaced such that they do not match the citation numbers in the reference section. The corrected **TABLE 1 | Derivation of human immunodeficiency virus (HIV) knowledge items with correct responses noted and descriptive statistics and TABLE 2 | Derivation of HIV-related stigma items and descriptive statistics** appear below. The authors apologize for this error and state that this does not change the scientific conclusions of the article in any way.

**Table 1:** Derivation of human immunodeficiency virus (HIV) knowledge items with correct responses noted and descriptive statistics.

Original items	Revised/final survey item	Total (*n* = 508)	% with correct response	Mean (SD) (mean scale of 0–1)	*p*-Value
When you think about HIV/AIDS, which group of people do you think of first as those who are most likely to be infected? And which group of people do you think of next? Gay men/homosexuals/bisexuals; drug users; teenagers/young people/promiscuous people, etc. (10)	Is it true that only homosexuals get HIV? **(No)**	508	99.4	0.99 (0.08)	0.48
Athletes who share needles when using steroids can get HIV from the needles (12)	Is it true that a person can get HIV by sharing drug needles? **(Yes)**	508	99.3	0.99 (0.10)	0.54
A person can get HIV by sitting in a hot tub or a swimming pool with a person who has HIV (11); a person can get HIV from a toilet seat (12); a person can get HIV by sharing a glass of water with someone who has HIV (12)	Can a person get HIV through casual contact with someone who has HIV, for example, by hugging, sharing a drinking glass, touching a toilet seat, or swimming in a pool? **(No)**	508	93.4	0.97 (0.17)	0.77
You can usually tell if someone has HIV by looking at them (12)	It is true that you can tell if someone has HIV or AIDS just by the way he or she looks? **(No)**	508	96.3	0.94 (0.24)	0.31
There are drugs available that can lengthen the lives of people who have HIV and AIDS (10)	If a person has HIV, are there drugs that can keep them healthy and alive for a long time? **(Yes)**	508	91.2	0.91 (0.28)	0.91
A pregnant woman who has HIV can take certain drugs to reduce the risk of her baby being born infected (10)	If a pregnant woman has HIV, are there drugs she can take to protect her baby from being born with HIV? **(Yes)**	508	71.1	0.66 (0.48)	0.01
There is a vaccine that can stop adults from getting HIV (11, 12)	Is there a vaccine available to protect people from getting infected with HIV? **(No)**	508	82.4	0.80 (0.40)	0.20
There are drugs available that can cure HIV and AIDS/there is a cure for AIDS (10, 12)	Are there drugs that can cure HIV and AIDS? **(No)**	508	86.8	0.82 (0.38)	0.003

**Table 2:** Derivation of HIV-related stigma items and descriptive statistics.

Original items	Revised/final survey item	Total (*n* = 508)	% with positive attitude	Mean (SD) (mean scale of 1–4)	*p*-Value
People who have AIDS are cursed (15)	I believe that HIV/AIDS is a curse from God **(Disagree/strongly disagree)**	503	94.0	3.53 (0.67)	0.02
A person with AIDS must have done something wrong and deserves to be punished (15)	I think that people who get HIV/AIDS did something to deserve it **(Disagree/strongly disagree)**	507	94.7	3.58 (0.64)	0.001
N/A	People with HIV/AIDS should get the same level of medical care as a person who has cancer or another type of chronic disease **(Agree/strongly agree)**	507	95.8	3.63 (0.65)	0.20
I hide my HIV status from others (16)/If a member of your family became infected with HIV, would you want it to remain a secret? (17)	It is better for the community if a person tells others if they have HIV **(Agree/strongly agree)**	507	80.9	3.29 (0.90)	0.20
Some people who have AIDS lose friends when they share with them they have AIDS (18)	If a friend of mine confided that they were HIV positive, I would cut them off. That friendship would be over **(Disagree/strongly disagree)**	508	96.4	3.68 (0.60)	0.002
If a person has AIDS, some community members will behave differently toward that person for the rest of his or her life (18)	If I told my best friend that I was HIV positive, my friend would not treat me any differently **(Agree/strongly agree)**	508	72.1	3.02 (0.99)	0.001
Some people who have AIDS worry that others will reveal their secret (18)	If I found out that I was HIV positive, it would affect my social life, even if I never told anyone **(Disagree/strongly disagree)**	507	23.7	1.88 (1.00)	0.48
I sometimes feel worthless because I am HIV positive (16)	If I tested positive for HIV, I do not think my life would change very much **(Agree/strongly agree)**	507	20.1	1.80 (0.94)	0.55
Some people who have AIDS are afraid that other people in the community will talk about them having AIDS (18)	I am afraid to talk about HIV because I wouldn’t want anybody to think that I have it **(Disagree/strongly disagree)**	508	92.0	3.42 (0.73)	0.14
If a member of your family became sick with HIV, would you be willing to care for him or her in your household? (16)	If I told my family that I had HIV, they would embrace me, support me and be there for me **(Agree/strongly agree)**	508	89.6	3.5 (0.76)	0.23
If a member of your family became sick with HIV, would you be willing to care for him or her in your household? (16)	If I told members of my church or religious group that I had HIV, they would support me and be there for me **(Agree/strongly agree)**	507	83.1	3.23 (0.86)	0.19
Some people feel uncomfortable being near those with AIDS (18)	If my brother or sister were HIV positive, I could never feel the same about them or be comfortable around them **(Disagree/strongly disagree)**	508	95.1	3.71 (0.62)	0.42
Some people prefer not to have those with AIDS living in their community (18)	If any of my neighbors were HIV positive, they should be forced to move somewhere else **(Disagree/strongly disagree)**	508	98.6	3.73 (0.51)	0.14

## Conflict of Interest Statement

The authors declare that the research was conducted in the absence of any commercial or financial relationships that could be construed as a potential conflict of interest.
